# Navigating the COVID-19 infodemic: the influence of metacognitive efficiency on health behaviours and policy attitudes

**DOI:** 10.1098/rsos.230417

**Published:** 2023-09-06

**Authors:** Matteo Lisi

**Affiliations:** ^1^ Department of Psychology, University of Essex, Essex, UK; ^2^ Department of Psychology, Royal Holloway, University of London, London, UK

**Keywords:** COVID-19, misinformation, metacognition, policy attitudes, truth judgements

## Abstract

The COVID-19 pandemic has been accompanied by an infodemic of misinformation and increasing polarization around public health measures, such as social distancing and national lockdowns. In this study, I examined metacognitive efficiency—the extent to which the subjective feeling of knowing predicts the objective accuracy of knowledge—as a tool to understand and measure the assimilation of misleading misinformation in a balanced sample of Great Britain’s population (*N* = 1689), surveyed at the end of the third national lockdown. Using a signal-detection theory approach to quantify metacognitive efficiency, I found that at the population level, metacognitive efficiency for COVID-19 knowledge was impaired compared with general knowledge, indicating a worse alignment between confidence levels and the actual ability to discern true and false statements. Crucially, individual differences in metacognitive efficiency related to COVID-19 knowledge predicted health-protective behaviours, vaccination intentions and attitudes towards public health measures, even after accounting for the level of knowledge itself and demographic covariates, such as education, income and political alignment. These results reveal the significant impact of misinformation on public beliefs and suggest that fostering confidence in accurate knowledge should be a key target for science communication efforts aimed at promoting compliance with public health and social measures.

## 1. Introduction

Poor adaptation of behaviours to recommendations based on accurate information and scientific evidence can have detrimental public health consequences. The COVID-19 pandemic has been accompanied by an ‘infodemic’ [1,2]—a rapid spread of false or questionable information, growing and evolving in parallel with the epidemic [3]. While it is known that misinformation can influence people’s beliefs and behaviours, quantifying the nature and extent of this influence remains challenging in practice [4]. Characterizing and understanding the effects of the infodemic on citizens' beliefs and behaviour is particularly important during the outbreak of a new disease like COVID-19, as the public health response is, at least initially, mostly based on non-pharmacological interventions. Since these interventions rely on the adoption of protective behaviours in the population and compliance with health and social measures, the infodemic has the potential to seriously compromise their efficacy. One avenue for quantifying the effects of misinformation could be to examine metacognitive aspects of knowledge [5]. Misleading information may impact empirical measures of metacognitive sensitivity obtained from confidence judgements, which reflect the extent to which a person’s confidence in their knowledge matches the accuracy of their knowledge. Misinformation can decrease the confidence with which accurate statements are accepted or increase the confidence with which misleading claims are rejected [6,7]. These effects may have important consequences on behaviour, as confidence is used to decide when to seek new information [8], guide future decisions [9], learn when immediate feedback is not available [10] and decide how much to learn from past rewards [11]. An accurate sense of confidence is associated with good decision-making across many domains [12–14]. Using an approach grounded in signal detection theory [15], a recent study provided evidence linking misinformation and metacognition, in the context of public assimilation of climate-change science [5]. In the study, Fischer and colleagues asked German citizens to judge the truth of statements about climate-change science or general science and rate the confidence in their answer. Confidence ratings were less predictive of accuracy when statements referred to climate-change science than general science, suggesting that misinformation impairs the ability to assess the (un)certainty of knowledge and judge when information is more or less likely to be correct.

In the present study, I estimate Great Britain’s population’s metacognitive insight into their knowledge of COVID-19, compare it with their insight into general science knowledge, and examine its relation to health protective behaviours. The pre-registered hypothesis tested is that British people’s insight into their knowledge of COVID-19 would be impaired compared with other areas of knowledge, replicating Fischer *et al.*’s [5] findings in a different domain and population. The study thus aims to bring additional support to the notion that the effect of misinformation on beliefs can be quantified by measures of metacognitive sensitivity. Additionally, I investigated whether metacognitive insight into COVID-19 knowledge influenced attitudes and compliance with public health and social measures during lockdown. To address these questions, I surveyed 1689 respondents, a nationally balanced sample of Great Britain’s population, in April 2021 at the end of the third national lockdown, asking them to indicate the truth of 28 statements about COVID-19 and general biological and physical sciences and rate their confidence in their answers. Respondents were also asked about their self-reported behaviours and attitudes. To anticipate the results, I found that British people’s metacognitive insight about the accuracy of their COVID-19 knowledge was less reliable than in other areas and that individual differences in metacognitive insight were predictive of attitudes, behaviours and vaccination intentions even after accounting for other factors (such as age, education, income or political alignment). These findings suggest that metacognitive insight can reveal the assimilation of misinformation, which influences attitudes and behaviours. Therefore, the accuracy of metacognition can both measure the impact of misinformation and offer a target for interventions aimed at mitigating such an impact.

## 2. Results

### 2.1. Knowledge accuracy and confidence level

**Figure 1.**
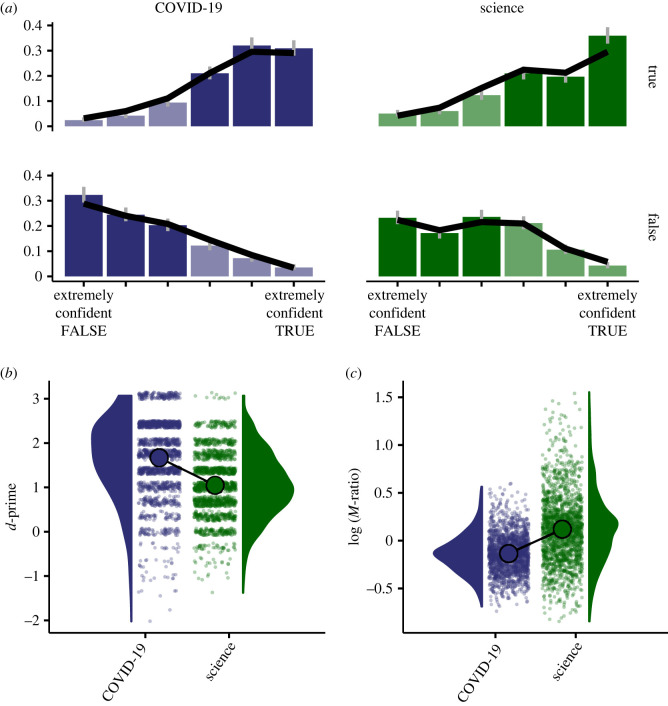
Confidence and knowledge accuracy in COVID-19 and general science. (*a*) Distributions of confidence ratings, split by category (COVID-19 versus general science) and classification of the statements (true versus false). The black lines represents the mean predictions of the multilevel model used to estimate metacognitive efficiency; the grey error bars are multinomial 95% CI computed on pooled data. Light and dark shading indicate wrong and correct answers, respectively. (*b*) Knowledge accuracy, as measured by the signal detection sensitivity index *d*′; accuracy was on average higher on COVID-19 statements. Small dots are individual participants (some jitter has been added) and the large dots represents the averages. (*c*) Metacognitive efficiency, quantified as the log (*M*-ratio), or log ((meta − *d*′)/*d*′); same conventions as in *b*. Despite the higher *d*′ observed in COVID-19 statements, metacognitive efficiency was higher for general science statements, indicating a better alignment between participants’ confidence levels and their actual ability to discern between true and false statements.

The data indicate that respondents were more frequently accurate at accurately judging the COVID-19 statements in the survey compared with the general science statements (figure 1*b*). Using signal detection theory to quantify their ability to discern true from false information, I found that the average sensitivity index *d*′ was 1.67 for COVID-19 statements, 95% CI [1.62, 1.72]; and 1.05 for general science statements, 95% CI [1.01, 1.09]. Additionally, respondents tended to report slightly higher confidence ratings for COVID-19 items. A Bayesian ordinal regression model (see electronic supplementary material, Results for details) showed that, on average, respondents were 1.05 times more likely to report higher confidence ratings for COVID-19 statements than for general science statements, with a 95% Bayesian credible interval of [1.00, 1.11] (highest posterior density interval, HDI). The higher accuracy of judgements about COVID-19 statements indicates that the difficulty of statements in these two conditions was not perfectly matched. In order to better understand metacognitive insight in the two domains while controlling in the best possible way for the difference in accuracy, I used a model-based approach (see next section).

### 2.2. Metacognitive insight

To assess metacognitive insight, I used a method based on signal detection theory [15]. This involves measuring the respondents’ sensitivity, or their ability to distinguish true and false statements, and expressing it in signal-to-noise ratio units (*d*′). Confidence ratings are then used to estimate metacognitive sensitivity (meta-*d*′), which represents the information available for confidence ratings in signal-to-noise units and provides a measure of how well confidence ratings can predict accuracy. One challenge in measuring metacognitive sensitivity is that it is bounded by sensitivity (*d*′), and therefore must be interpreted in relation to it [15,16]. To quantify metacognitive insight, I used the *M*-ratio, which is the ratio of meta-*d*′ to *d*′. An *M*-ratio of 1 would indicate that a respondent’s confidence ratings are as informative as possible about the accuracy of their judgements. A value smaller than 1 instead would suggest a loss of information. For example, an *M*-ratio of 1/2 would indicate that the same level of informativeness of confidence ratings could have been achieved with only half the knowledge, suggesting an impairment of metacognitive insight.

**Figure 2.**
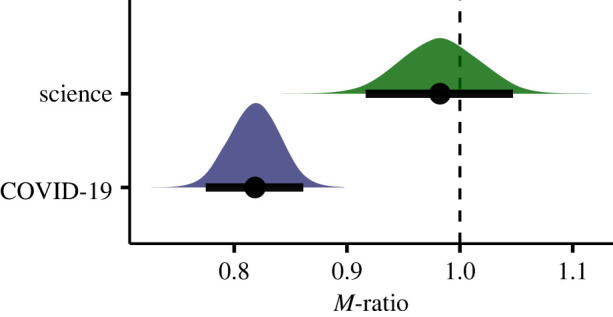
Population-level estimates of metacognitive efficiency. Posterior distributions of the fixed-effects estimates of the metacognitive efficiency (*M*-ratio) parameter for COVID-19 and general science; the dots represent posterior mean and the black lines indicate 95% HDI (highest posterior density) intervals.

I estimated *M*-ratio values using a multilevel Bayesian approach [17]. Figure 1*a* shows the distribution of confidence ratings, separately for all conditions, together with the fixed-effect predictions. Figure 2 shows the posterior distributions of population-level (fixed-effects) estimates of metacognitive efficiency (*M*-ratio) for COVID-19 and general science. Overall, this analysis revealed that at the population level the metacognitive efficiency was estimated to be 0.98, 95% CI [0.91, 1.05] (highest posterior density interval, HDI) for general science, and 0.82, 95% CI [0.77, 0.86] for COVID-19. The estimated Bayes factor indicates that our data are approximately 147 times more likely under the hypothesis of a difference in *M*-ratio across the two domains of knowledge than under the (point) null hypothesis, supporting the pre-registered hypothesis.^1^ Because the *M*-ratio was smaller for judgements about COVID-19 statements, the results indicate an impairment in metacognitive insight specifically into COVID-19 knowledge. Furthermore, supplemental analyses suggested that this difference was not driven only by a minority of respondents that explicitly subscribed to COVID-19 conspiracy theories: nearly identical results were obtained after excluding participants (18.77% of total sample) who as true judged the statement ‘COVID-19 is a bio-weapon intentionally spread by the Chinese state to weaken Western economies’ (see electronic supplementary material, §4.2). Next, I examined to what extent individual differences in metacognitive efficiency for COVID-19 knowledge can predict vaccination intentions as well as behaviour and attitudes during lockdown.

### 2.3. Metacognitive insight predicts attitudes and behaviour during lockdown

**Figure 3.**
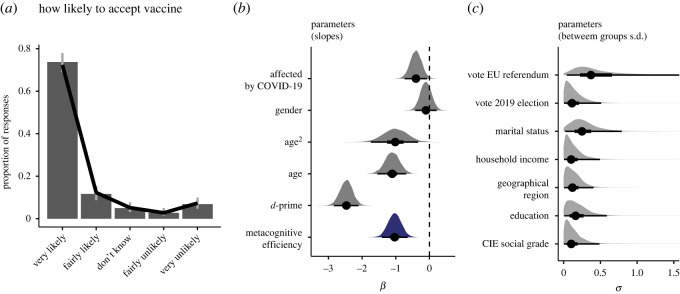
Association between metacognitive insight and vaccine intentions. Ordinal regression analysis, used to evaluate the relationship between metacognitive insight and vaccination intention (see Methods for details of the approach). (*a*) Distribution of responses (grey error bars are multinomial 95% CI); the black line represents the predictions of the ordinal regression. Note that this analysis was run excluding the 36% of the respondents who declared to have already received at least one dose of the vaccine. (*b*) Posterior distribution over model parameters (slopes). The dots are posterior means and the horizontal lines indicate 50% and 95% HDI intervals. Negative slope values indicate that for each increment in the predictor the probability mass moves from categories that are represented on the right in the histogram in *a* (e.g. ‘very unlikely’) towards categories on the left (e.g. very likely). The variables ‘gender’ and ‘affected by COVID-19’ were represented as binary dummy variables (‘affected by COVID-19’ was set to 1 if respondents answered positively to the question of whether they knew of anybody, including themselves, whose health had been badly affected by COVID-19. Metacognitive efficiency was quantified as the logarithm of individual *M*-ratio estimates. For this analysis continuous predictors were scaled by 2 s.d. (see Methods). (*c*) Posterior distribution of standard deviations of random effects, for the demographic variables and other relevant covariates included in the model; the larger the standard deviation, the larger the differences between groups (e.g. income bands, geographical regions etc.). See electronic supplementary material, figure S17 for a plot of posterior distribution of group-specific intercepts. Overall, this analysis indicates that metacognitive efficiency carries useful information for predicting individual vaccine intentions even after taking into account all the other covariates and demographic characteristics.

**Figure 4.**
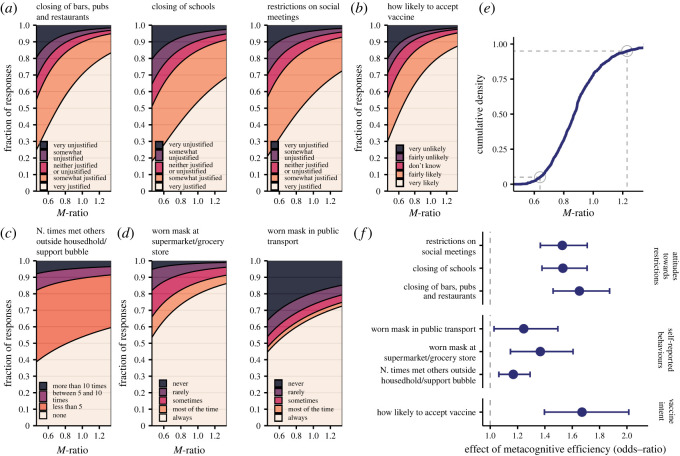
Metacognitive insight is associated with positive attitudes, behaviours and vaccination intention. Marginal effects plots, showing how increasing metacognitive efficiency (*M*-ratio) is associated with changes in response probabilities are shown in (*a*) for attitudes towards restrictive measures, (*b*) for vaccination intentions, (*c*) for social meetings (socially meeting others outside of household or support bubble was forbidden during the lockdown) and (*d*) for mask-wearing. In each panel, the predicted response probabilities are shown along the vertical axis as the relative heights of shaded areas. These have been calculated holding all other predictors constant, and all random intercepts to zero. To contextualize values of *M*-ratio, (*e*) shows the cumulative distribution function of estimated *M*-ratio scores for individual participants (5% and 95% percentile are highlighted). Panel (*f*) shows the estimated effects of metacognitive efficiency (posterior mean with 95% HDI intervals) for each of the questions, expressed as odds ratio, that is the multiplicative change in the odds of respondents indicating more positive attitudes and compliant behaviours associated with an increase of 1 s.d. in metacognitive efficiency. For all questions, we found a reliable association of responses with metacognitive insight. (Note: a separate model was run for each question; see Methods for details).

After judging the truth of the 28 statements, respondents answered a series of questions about their attitudes and behaviour during the lockdown; one question about their vaccination intention and one question about whether their health (or the health of someone they knew) had been badly affected by COVID-19 (this was the case for nearly 40% of respondents; see electronic supplementary material, §4.5). The full set of questions is reported in electronic supplementary material, §1.3. I ran a series of Bayesian ordinal regressions to examine whether metacognitive insight (quantified as the logarithm of individual *M*-ratio scores) could predict their responses. In addition to metacognitive insight and knowledge (the *d*′ sensitivity index, which quantifies respondents' ability to discern true and false information), the models included several covariates as predictors (see Methods and figure 3*b,c*). Some of these covariates correlate with signal-detection theoretic parameters (see electronic supplementary material, §§4.5 and 4.6) and therefore we included them in the analyses to control for possible confounding effects. Continuous variables (e.g. age) or categorical variables with only two levels were included as fixed-effect predictors. The remaining categorical variables, which had more than two levels and could generally be understood as belonging to a particular group (e.g. married couples, or people living in London) were included as random intercepts. The analysis is illustrated in figure 3 with the example of the question about vaccine intentions. The histogram in figure 3*a* represents the proportion of responses: we can see that among those respondents who had not yet received one or more doses of the COVID-19 vaccine, nearly 75% indicated that they would be very likely to accept it. Figure 3*b* shows posterior distribution for the fixed-effect slopes, and figure 3*c* shows posterior distribution for the standard deviations of the random intercepts (representing the size of differences between groups). The pseudo *R*^2^ [18] for the model in figure 3 was 0.21. Crucially, the posterior distribution in figure 3*b* indicates that, even after taking into account all covariates (social, grade, income etc.), the metacognitive ability still carries useful information for predicting the responses. The marginal effect of metacognitive ability on vaccination intention, which illustrates how intentions are predicted to change across a plausible range of *M*-ratio values (holding constant all other predictors), is shown in figure 4*b*. In sum, this analysis thus indicates that the more accurate is people’s metacognition about their knowledge of COVID-19, the more likely they are to accept a vaccine. The same ordinal regression approach was used for all questions (see electronic supplementary material, §5.2, for detailed plots of each model). In all cases, we found a reliable effect of metacognitive ability (figure 4*f*), although there seems to be a tendency for the effect to be larger on attitudes and vaccine intentions than on self-reported behaviours. Figure 4*a*–*d* illustrates the marginal effects of metacognitive efficiency, calculated over a plausible range of *M*-ratio values (compare with the cumulative distribution function plotted in figure 4*e*) holding constant all other predictors. These results revealed that overall, across all questions, respondents with better metacognitive insight about their COVID-19 knowledge systematically reported more positive attitudes about and better compliance with restrictive measures. The effect was specific to metacognitive insight around COVID-19 knowledge: in a series of control analyses we included also metacognitive efficiency in general science as an additional predictor, but found that it was not reliably associated with compliance and behaviours in any of the questions (see electronic supplementary material, Results).

## 3. Discussion

The findings presented in this study demonstrate that metacognitive insight about COVID-19 knowledge, measured using a signal-detection theoretic approach, was impaired during the third national lockdown in Great Britain compared with general physical and biological sciences that arguably are less affected by misinformation. This mirrors previous findings on metacognitive climate-change knowledge in German citizens [5], although the impairment reported in the present study was not as large: respondents in our study achieved 82% metacognitive efficiency as opposed to 47% reported by Fischer and colleagues (whereas in both studies metacognitive efficiency in general science was not different from 100%). Importantly, I also found that individual differences in metacognitive insight about COVID-19 knowledge were predictive of attitudes towards restrictions, behaviours during lockdown and vaccination intentions—even after taking into account covariates such as the accuracy of knowledge itself, education, political alignment or income. These findings, which are in agreement with a recent study on a German sample [19], illustrate how metacognitive insight about one’s own knowledge can affect decision-making in areas relevant for public health and social measures, in agreement with prior research in other domains [20–22].

From a methodological standpoint, the results reported here support the use of metacognitive insight into relevant knowledge—measured by applying signal-detection theoretic models to confidence ratings of true/false choices—as a useful way to quantify the impact of misinformation on beliefs. Compared with simple dichotomous choices or Likert scales of agreement, this methodology offers advantages, particularly in enabling the independent assessment of metacognitive efficiency [15] and knowledge accuracy, thereby providing further depth to conventional analyses. The metacognitive efficiency measure proves especially useful in assessing misinformation as it does not merely signal outright acceptance of misleading information as true and/or beliefs in far-fetched conspiracy theories. Instead, it can reflect more subtle effects that may occur if misinformation makes accurate beliefs appear less plausible, or makes misleading claims appear less implausible (by decreasing confidence with which we judge them as true or false, respectively). Furthermore, increasing belief in one piece of misleading information is bound to have effects that spill over to other, related beliefs. Indeed, it has been argued that subjective knowledge assimilates new information as a ‘corporate body’ [23,24] rather than as a set of disconnected beliefs, a notion supported also by empirical research showing how subjective beliefs about the world are not updated in isolation in the face of new evidence [25,26]. This implies that probing the strength of beliefs in a set of statements via confidence judgements may reveal the influence of misinformation that is not explicitly represented in the set. Overall, these considerations suggest that even subtle and indirect influences of misinformation may be detected using this method, as long as the misinformation moves the whole network of subjective beliefs away from accurate knowledge.

Psychological research has shed some light on the cognitive mechanisms that can promote people’s belief in a claim or news information regardless of its truth [4,27]. For example, repeated exposure to a statement has been shown to increase later subjective truth ratings [28–32], an effect that seems to be independent of either the plausibility of the statement [33] or individual differences in cognitive ability [34], and that persists even when the information is explicitly labelled as contested by fact-checkers [35]. Thus, repeated exposure to misinformation may influence people’s beliefs and have cascading effects on attitudes and behaviours [36]. Some recent studies have experimentally manipulated exposure to misinformation and reported that even a single exposure had measurable effects on behavioural intentions [37,38]. Although the effects of single exposures in these studies were relatively modest, the real-world effects of many, frequent exposures are difficult to quantify and should not be underestimated, especially considering the ubiquity of misleading claims in the current information environment. For example, in the UK, misleading information about COVID-19 has been broadcasted even by public officials: in November 2020, at a time when recorded deaths were 14% higher than the 5-year average according to the Office for National Statistics, a Member of Parliament claimed in an interview that official COVID-19 statistics were being manipulated and that the number of deaths did not exceed typical levels for that period of the year [39]. Repeated exposure to such misleading information may not only make the specific statement (e.g. ‘death statistics are being manipulated’) appear more believable but may also influence other related beliefs—for example, by decreasing the confidence that related statements such as ‘COVID-19 is a greater health threat than influenza’ are true. I suggest that these pervasive, subtle influences are what underlie the differences in metacognitive insight reported here. Indeed, I find that the difference in metacognitive efficiency between COVID-19 and general science remains virtually unchanged even after removing the subset of participants (approx. 19% of total) that are arguably most likely to believe in conspiracy theories—i.e. those who judged as true the claim that COVID-19 is a bio-weapon devised by the Chinese government (see electronic supplementary material, Results).

There are some caveats to consider in interpreting the current findings. As COVID-19 is a relatively new disease, there is greater epistemic uncertainty around several of its features compared with other topics. It is important to note that accurate information around COVID-19 was nevertheless already available at the time of the survey, and when selecting the true statements, I carefully sought to avoid statements that could not be backed by evidence (see electronic supplementary material). However, if respondents were influenced by the epistemic uncertainty around COVID-19, they could have been biased against reporting high confidence ratings for judgements about COVID-19 statements, and this could have negatively affected their metacognitive efficiency estimates. Contrary to this explanation, though, I found that participants tended to report slightly higher confidence ratings in their judgements of COVID-19 statements than general science ones, even if accuracy was controlled for (see electronic supplementary material, Results for details). Thus, the data does not provide evidence for reduced confidence in COVID-19 knowledge, and instead suggests that respondents may even overestimate it, possibly as a result of familiarity with information related to COVID-19 circulating during the pandemic [28].

Another consideration is the potential influence of social desirability bias. Although the survey was conducted online with confidentiality ensured, we cannot completely rule out that social desirability had some influence on participants’ responses. Pools conducted prior to the survey indicated that a majority of population supported the restrictions (e.g. [40]) and it is thus possible that social desirability inflated compliance and support for the restrictions in our dataset.

Furthermore, the specific statements used in the study may have influenced the relationship between metacognitive efficiency estimates (*M*-ratio) and beliefs in public health messages. At the time of the survey, it was difficult to find accurate statements that discouraged health-protective behaviours. As a consequence the truthfulness of the statements is correlated with being promotive of health protective behaviours (and therefore also with public health messages). While this does not undermine the utility of the *M*-ratio as a summary measure of misinformation impact, it does limit the ability to disentangle whether the results are driven solely by belief content or by impairments in individuals’ meta-cognitive processes. To provide a preliminary answer, I examined correlations between individual COVID-19 questions and self-reported behaviours and attitudes (see electronic supplementary material, §4.7). Overall, the analysis revealed not only evidence for expected correlations (based on contents of individual statements), but also for correlations that are not directly following from the statements’ content. For instance, the belief that COVID-19 vaccines may alter DNA was associated not only with lower vaccination intentions but also with negative attitudes towards restrictions and social distancing measures. Taken together, these suggest that the content of beliefs interacts with individuals’ broader belief system and cognitive and meta-cognitive factors to shape behaviours and attitudes.

One limitation of the present study is that due to its observational nature, it does not provide direct evidence about the direction of causal relationships. While it seems reasonable to assume that misinformation may cause the impairment in metacognitive insight and consequent reductions in health-protective behaviours as effects, in principle the causality could be reversed: people with better metacognitive ability may be less influenced by misinformation. Indeed, some studies have reported that lower metacognitive ability is associated with holding radical beliefs and displaying tendencies towards polarization and dogmatism [41–43]. Based on this interpretation, one would expect that health-protective behaviours be associated also with metacognitive insight in general science, and not just with metacognitive insight specifically around COVID-19 knowledge. However, repeating the regression analyses including metacognitive efficiency in general science as a predictor revealed that in all cases, only metacognitive insight in COVID-19 knowledge predicted attitudes and behaviours (see electronic supplementary material, Results). This analysis thus supports the notion that the signal-detection theoretic measure of metacognitive insight genuinely reflects the extent to which exposure to misinformation (which may vary largely across individuals) has affected individual beliefs, attitudes and behaviours.

In conclusion, our results suggest that to promote compliance with public health measures as effectively as possible, science communication should strive to improve the confidence and metacognitive insight of the population in the evidence that guides public health strategies. While persuasion and science communication are already recognized by behavioural scientists as priorities for promoting compliance [44], it remains unclear which approaches and practices are most effective. Common approaches to debunking misinformation, involving corrections and fact-checking, may reduce belief in misleading information [45,46], but it has been suggested that these approaches may also backfire [47,48]—although the evidence around this is mixed [49,50]. In addition to reducing belief in misleading information, our results suggest that it is also important to promote belief in accurate information. Prior research indicates that this may benefit from increased scale and frequency of messaging [51] and by enlisting credible sources that are perceived as trustworthy by the public [44]. Promoting confidence in accurate beliefs also presents challenges: for example, it remains unclear how to best communicate the epistemic uncertainty around scientific evidence [52–54]. Ultimately, more empirical research is needed to determine which type of communications and interventions are more effective at combating misinformation and promoting beliefs in accurate knowledge and compliance with public health measures. Given the systematic associations shown here between metacognitive insight around COVID-19 knowledge and attitudes and behaviour, I suggest that the methodology used here to measure metacognitive insight may provide a promising avenue to evaluate the effectiveness of different approaches.

## 4. Methods

The survey was carried out online; the data were collected by YouGov Plc (www.yougov.co.uk). Respondents (*N* = 1689 adults) were members of the YouGov GB panel, comprising more than 185 k individuals who have agreed to take part in surveys. An email invitation, containing the link to the survey, was sent to panellists selected at random from the base sample according to balanced quotas for age, gender, social grade, geographical location and political preferences in Great Britain. The final sample contained some discrepancies for the target quotas; see electronic supplementary material, table S2 for a detailed breakdown of respondents’ characteristics and a comparison with GB population. The data were collected between 13 and 14 April 2021, immediately after the beginning of the gradual easing of lockdown restrictions: Monday 12 April was the first day of opening of non-essential retail [55] since the start of the third national lockdown on 6 January. Ethical approval for this study was provided by ethics committee of the Department of Psychology of the University of Essex (reference ETH2021-1153).

### 4.1. Materials

#### 4.1.1. General science knowledge

Following Fischer *et al.* [5], we selected eight statements from the factual knowledge questions of the National Science Foundation [56] and included six new statements in order to reach a total of 14 statements, equally split between true and false (see electronic supplementary material). These statements were chosen based on their expected stability over time, allowing for comparisons with previous and future studies. While they do not encompass the full spectrum of scientific subjects, they serve as an indicator of individuals’ engagement with science throughout their lives [57], providing insights into their familiarity with non-controversial and non-politically polarized scientific ideas.

#### 4.1.2. COVID-19 knowledge

While for general physical and biological science identifying factually correct and wrong statements is relatively straightforward, the task is more complex for statements about COVID-19, and particularly so for true statements since COVID-19 is a relatively new disease, and the scientific evidence around it is evolving quickly. Nevertheless, basic facts that can be accepted with high confidence are also available in the case of COVID-19 (and related topics, such as vaccine or face masks), and have been used to guide the public health response. For instance, these include information such as the incubation period of COVID-19 (that is the average temporal interval between infection and onset of symptoms) or that the virus may be transmitted by people who do not yet show any symptoms. We selected a series of statements that reflected the knowledge available in early 2021, and should also be contextualized to that phase of the pandemic (therefore the statements implicitly refer to the original SARS-CoV-2 virus and variants of the alpha and beta lineage, but not variants of concern that emerged later). Similar to the general science statements, the COVID-19 statements were also selected based on their expected stability over time. We included also statements that referred directly to misinformation and conspiracy theories around COVID-19 (e.g. that the virus was designed as bio-weapon, or that COVID-19 vaccines can affect fertility). In total, we selected 14 statements, equally split between true and false (see electronic supplementary material for the full list of statements and the sources or rationale that informed the classification of each statement as true or false). Although these statements touch upon a wide range of distinct aspects of the COVID-19 pandemic, taken together they achieved an acceptable level of internal consistency (Crombach’s *α* = 0.75), suggesting that the knowledge to answer them correctly was correlated across respondents.

#### 4.1.3. Attitudes and self-reported behaviours

After judging the 28 statements, participants answered a set of five questions that focused on their behaviours during the lockdown, their attitudes towards the restrictive measures, vaccination and their personal experience with COVID-19 (see electronic supplementary material).

### 4.2. Procedure

Respondents first judged the accuracy of the 28 statements about COVID-19 and general science, randomly interleaved across individuals. Respondents judged the accuracy of the statement and the confidence in their response by selecting a response on a 6-point scale: (1) ‘I am extremely confident that this is true’; (2) ‘I am fairly confident that this is true’; (3) ‘I think this is true, but I am not at all confident’; (4) ‘I think this is false, but I am not at all confident’; (5) ‘I am fairly confident that this is false’; (6) ‘I am extremely confident that this is false’. We only used three levels of confidence because there is evidence that human intuition represents subjective probability only with a limited precision [9,58], so increasing the granularity by adding extra confidence levels (with a corresponding increase in the number of parameters to be estimated in the signal detection theory model) is unlikely to provide more information about their metacognitive ability. We also did not use an explicit numerical probability scale because respondents’ intuitive understanding of probabilities is likely to introduce distortions [58,59], which would complicates the interpretation of any deviations from objective probability as genuine overconfidence or underconfidence biases. After judging the truth of these 28 statements, respondents proceeded to answer the questions about attitudes and self-reported behaviour, which were presented in a fixed order (see electronic supplementary material).

### 4.3. Analysis

#### 4.3.1. Estimation of metacognitive ability

Metacognitive ability was quantified using a signal-detection theoretic approach [15]. The model was estimated using a multilevel Bayesian approach [17] implemented in Jags [60] via its R interface [61,62]. The model was estimated by running three chains for 30 k samples, after 5 k burn-in samples, with thinning of 9, yielding 9999 total posterior samples. Convergence was verified by visual inspection of the chain and by checking that all *R*-hat values were less than 1.01 [63]. Our approach is similar to that of Fischer *et al.* [5], with the difference that in our case the same sample respondents judged the truth of statements on two areas (general science and COVID-19, randomly interleaved), so we modified the jags code to fit the within-subject design. One further difference is that to improve the estimation given the low number of judgements per condition, and to estimate a Bayes factor, we used an informative prior on the population-level metacognitive efficiency parameters. See electronic supplementary material, Results for convergence statistics and the details about the prior choice and the calculation of the Bayes factor.

#### 4.3.2. Estimating associations between metacognitive efficiency, attitudes and self-reported behaviours

In order to measure association between metacognitive insight and the responses to questions about attitudes and behaviours, we used an ordinal regression model. The details of this approach are reported in the electronic supplementary material, Methods; in brief, this model corresponds to a Bayesian multilevel ordered logit model [64]. The predictors included in this analysis were: *d*′, log *M*-ratio, age, gender, chief income earner (CIE) social grade [65], geographical region, vote at the 2019 general election and at the EU referendum, education level, marital status and personal income. Continuous predictors (age, *d*′ and log *M*-ratio) were centred and scaled by 2 s.d. to put them approximately on the same scale as the other dichotomous dummy variables [66]. All fixed-effects parameters (slopes) were given standard normal distribution as priors. The remaining variables that were categorical and had more than two levels were included as random intercepts, such that also the standard deviation between groups was explicitly estimated; these were given HalfCauchy priors, with location and scale parameters set to 0 and 1, respectively. For the cut points in the latent space, we induced a regularizing prior by setting a flat Dirichlet prior on the ordinal probabilities and pushing this forward to the latent cut points [67]. The models were estimated in Stan [68], via its R interface [69]. For each model, we run four chains with 4 k samples each (2 k burn-in); to check convergence we verified that there were no divergent transitions and that *R*-hat was less than 1.01 for all parameters. Note that the demographic information such as income or education was not asked within the present survey: respondents provided this information as part of their registration process as online panelists. Some of the variables had missing values: 26 respondents (1.5%) skipped or were not asked the question about vote in the 2019 general elections; 31 respondents (1.8%) indicated that they did not remember what they voted in the EU referendum; 79 respondents (4.7%) skipped or were not asked the question about marital status; finally, regarding personal income, 73 respondents (4.3%) answered ‘don’t know’, 328 respondents (19.4%) answered ‘prefer not to say’, and 95 respondents skipped or were not asked the question (5.6%). In order to retain all the data in the analyses, we added extra categories to each variable to indicate the missingness (see electronic supplementary material, figures for details for plots showing posterior distribution and credible interval of all group-specific intercepts for all models). The education level was recoded into a smaller number of categories (see electronic supplementary material, table S1).

## Data Availability

Data and code supporting this article are available as an Open Science Framework repository link: https://osf.io/nd9yr/ [70]. The data are provided in electronic supplementary material [71].

## Footnotes

^1^
https://doi.org/10.17605/OSF.IO/CU8ZQ.
